# Advances in Amplification Methods for Biosensors

**DOI:** 10.3390/bios13030365

**Published:** 2023-03-10

**Authors:** Arnaud Buhot

**Affiliations:** Grenoble Alpes University, CEA, CNRS, IRIG-SyMMES, 17 Rue des Martyrs, 38000 Grenoble, France; arnaud.buhot@cea.fr

Today, there is a rapidly growing demand for sensitive and selective biosensors in various domains, including environmental monitoring such as (waste)water control, detection of pollution for personal/public safety, agricultural/food safety and quality control, veterinary and medical diagnostics, etc. For these applications, the main challenge remains to detect a minute amount of analytes generally present in complex samples. Thus, recent biosensors based on the biomolecular recognition between analyte targets and relevant probes (either antibodies, aptamers, or molecular imprinted polymers) require the use of amplification methods to produce a measurable signal.

The Special Issue “Advances in Amplification Methods for Biosensors” aims to highlight the recent advances in the design of probes coupled to the development of innovative amplification methods in order to improve the performance of biosensors.

The COVID-19 pandemic is a perfect demonstration of the need for nucleic acids amplification test (NAAT) in the detection of SARS-CoV-2 virus variants but also for other circulating viral pathogens. In this context, the review by Akarapipad et al. [1] illustrates the importance of amplification methods, such as real time polymerase chain reaction (RT-PCR), loop-mediated isothermal amplification (LAMP), or recombinase polymerase amplification (RPA) for the multiplex detection of nucleic acids. For the Point-of-Care or *on field* detection, NAAT requires the integration in devices with the ASSURED criteria (Affordable, Sensitive, Specific, User-friendly, Rapid, Equipment-free, and Deliverable to end-users) set by the World Human Organisation twenty years ago [2]. The paper by Lan et al. [3] reports on a significant step towards low-cost and multiplexed RT-PCR device for on-site testing investigations. For less than a thousand dollars, this apparatus may perform rapid and sensitive testing of diverse diseases and is perfectly adapted for developing countries.

In the post-COVID years, isothermal amplification methods, and LAMP in particular, emerge among the NAAT as a competitive alternative to the standard PCR due to their ease of integration in portable devices. While the (nearly) doubling of nucleic acids strands per each PCR cycle easily explains the exponential amplification of targets, little is known in the evolution kinetics of LAMP products. Savonnet et al. are tackling this question and provide a simple kinetic model in their paper [4] along with an experimental validation of the relevant parameters. Those results could help improve the standard detection of viral or pathogen genomes, but also of miRNA targets recently emerging as important biomarkers in multiple diseases.

NAAT are not limited to the detection of nucleic acid targets but it may also allow for protein detection through the use of aptamers as probes [5]. In their paper [6], Jauset-Rubio et al. demonstrate another interesting use of the nucleic acid nature of aptamer probes. They take advantage of the PCR amplification to incorporate multiple biotins inside the aptamer probes through the use of biotinylated dUTPs. Thus, their Enzyme-Linked Aptamer Assay (ELAA) for the detection of allergen protein β-conglutin presents impressive sensitivity (sub-picomolar limit of detection) thanks to the multiple binding of streptavidin-polyHRP onto the aptamers.

The team of Prof. F. Seidi considers the use of surface modifications to enhance the probe densities leading to improved biosensor performances. In both studies, they considered electrochemical sensors with glassy carbon electrodes covered by β-cyclodextrins through electro-polymerization. In the first study, dendritic fibrous nano-silica particles were decorated by antibodies and deposited onto the electrodes. The high surface coverage of antibodies brought by the nano-particles ensures the sensitive detection of α-synuclein, a Parkinson’s disease biomarker [7]. In the second study, gold nano-particles are grafted on the surface to serve as a signal amplifier in sandwich-type immunno-sensors for the detection of the endocannabinoid 2-AG [8].

Finally, Lei et al. [9] demonstrate the detection of the small molecule forchlorfenuron, a plant growth regulator, thanks to the development of a sensitive and selective monoclonal antibody. Their indirect enzyme linked immuno-sorbent assay (ELISA), as well as colloidal gold nanobead immuno-chromatographic strip tests, presented interesting performances directly on cucumber samples.

While this first edition of “Advances in Amplification Methods for Biosensors” presents several aspects of up-to-date research in the field, further relevant aspects are expected in the second edition [10]. All the potential analyte targets may be considered, ranging from molecules (ions, pesticides, hormones, antibiotics, endocrine disruptors, miRNA, proteins, biomarkers, etc.) to larger objects (viruses, spores, fungus, bacteria, cancer cells, etc.). Biosensors based on optical, electrochemical, chemiluminescence, fluorescence, resonant or mechanical transduction methods are encouraged. Finally, the amplification methods are not restricted: nucleic acid amplification (PCR, LAMP, RPA, Rolling Circle Amplification, Hybridization Chain Reaction, etc.) or logic gate circuits, enzymatic amplification (enzymes, DNAzymes, CRISPR, etc.), nanostructure-based amplification (nano-particles, nano-tubes, nano-vesicles, MEMS/NEMS, nano-sensors, etc.), as well as combined strategies implying several amplifications.
